# History of a Girl with an Extraordinary Conformation of the Heart, with Observations on Animal Heat, Illustrative of the Case

**Published:** 1802-12-01

**Authors:** Martin Tupper

**Affiliations:** Exeter College, Oxford


### *History of a Girl with an extraordinary Conformation of the Heart, with Observations on Animal Heat, illustrative of the Case;* by MARTIN TUPPER, *of Exeter College, Oxford.*

MARIA BAILEY, ætat. 13, was rather ſtout of her age, and of middling ſtature, had a ſcrophulous aſpect, a thin cuticle, and light brown hair; ſhe appeared perfectly well nouriſhed, but her whole ſkin was always more or leſs of a bluiſh purple colour, more particularly the face, lips, and extremities of the fingers.

She was born at the full period, when it was obſerved by the parents that her ſkin was not ſo red as it is uſually at that time. She was unuſually quiet for the firſt fortnight of her life, but ſoon afterwards, and without any evident cauſe, ſhe became troubled with almoſt inceſſant fits of crying, during which the expirations were ſo long continued and violent that they frequently threatened ſuffocation, and induced a very great degree of blackneſs in the countenance; the ſucceeding inſpirations were always very difficultly performed, and were accompanied by a ſtridulous ſound. Theſe diſtreſſing ſymptoms continued very violent for the ſpace of five months. She was always worſe in the night time, or when ſhe laid in a recumbent poſition; ſhe often dozed in the day, but her ſleep was much diſturbed by ſighs and efforts to cry, and it ſcarcely ever was complete.

At the age of five months ſhe was put under the management of a nurſe in a very airy ſituation at Clapham, where ſhe remained a year and a half, during which the crying fits ſtill continued very frequent and violent.

At two years old, (having been weaned about four months) ſhe was ſent home; the crying had at that time in ſome degree diminiſhed, but a cough had begun to diſturb her; and it was obſerved, as a circumſtance which had not been before noticed, that the blue colour remained in the intermiſſion of the fits.

At the age of eighteen months, (the period when ſhe began to walk) it was obſerved that a great difficulty of breathing took place during, and remained ſome time after, any exertion.

Soon after this, the parents ſaw with concern that ſhe could not run and play with other children without being threatened by ſuffocation.

At ſeven years of age the patient returned to London. Her body was throughout of a purpliſh colour, but more particularly her lips, face, and arms. Her reſpiration continued to be very much affected, and the heart diſturbed in its actions, by the leaſt exertions. She was always very chilly, and generally ſought the fire even on the hotteſt ſummer's day. Her appetite was good, and the urine and ſtools perfectly natural. Her ſleep was tolerable, provided her head was well propped up, but it was accompanied by a rattling noiſe in the throat. She always laid on the back, with her head thrown much backwards, and her mouth open. She often ſtarted while aſleep, and very often (her head having either ſlipped too low, or having deviated from the poſition juſt mentioned) the rattling in the throat became exceſſive, when ſhe ſtarted up in great fright, much ſwoln in the countenance, and threatened by ſuffocation.

She never perſpired under any degree of exerciſe. She always felt herſelf cold during and ſome time after any exertion, although increaſed frequency of pulſe and of reſpiration were always the conſequence. When ſhe walked in cold weather, ſhe did not haſten her pace to increaſe the temperature on the ſurface, but, on the contrary, was in the habit of ſtanding ſtill for a while to produce that effect.

Having continued in this ſtate (though rather getting worſe and worſe every winter) till the autumn of 1799, the difficulty of breathing, cough and ſtartings during ſleep became exceſſive. The actions of the heart were frequently much diſturbed, and the difficulty of breathing was proportioned to their violence. She was in ſome degree relieved of theſe ſymptoms by bliſters, &c. From this period till November, 1800, ſhe continued nearly in the ſame ſtate, when ſhe was admitted an out-patient at Guy's Hoſpital. Dr. Babington obſerved, that her various ſymptoms, together with her general appearance, aroſe from ſome ſingular conformation of the heart, which no medicine could poſſibly remedy; he however preſcribed a bliſter for the ſcrobiculus cordis, and the infus. roſæ. c. magnes. vitr. to remove a coſtiveneſs which ſhe then laboured under. When the temperature of the ſurrounding medium was at 42° of Fahrenheit, I meaſured her animal heat; the mercury under her tongue then ſtood up to 96°; her pulſe beat 82 in a minute, and was rather ſmall and quick; her reſpiration was rather uneaſy; her face, lips, and fingers, and her whole body were as uſual of a purpliſh hue. Half an hour afterwards I repeated the experiment with the ſame reſult. I lament that I had no opportunity to make further obſervations upon this curious ſubject.

The patient continued much the ſame till February 18, 1801, when ſhe gradually became unuſually heavy and ſleepy. Two or three days after this, ſhe got out of bed in the middle of the night, to alleviate an inordinate thirſt, by which ſhe had been troubled troubled ſome days; and at about ſix o'clock the following morning ſhe expired, without the leaſt ſtruggle or ſign of ſuffocation.

She had never been ſuſceptible of fear, and was very little affected by the paſſions of the mind. Whether the atmoſphere was warm or cold, damp or dry, ſhe always felt equally chilly. She was not more partial to animal than to vegetable food, or the contrary. Her appetite, urine, and ſtools, were perfectly natural. She had never menſtruated.

### Appearances on Dissection.

The lungs were very ſmall, and receded very far back, ſo as to appear puckered up towards the ſpine. There was rather more ſerum collected in the right ſide of the pleura than uſual.

Upon opening the pericardium, the heart appeared natural, and together with its veſſels was perfectly in ſitu.

The liquor pericardii was natural and in due quantity.

The liver was rather larger than natural.

The ſpleen was rather ſmall, though not more ſo than it is ſometimes met with.

The alimentary canal was perfectly ſound, except the great arch of the colon, which was præternaturally contracted.

The cellular membrane was œdematous.

The muſcles were as florid as they generally are.

Nothing was to be obſerved with reſpect to the capacity of the auricles or ventricles; but, upon examining the former, the foramen ovale appeared in the ſame ſtate as it is in the fœtus. There was likewiſe found a deficiency in the ſeptum ventriculorum at the baſis of the heart, and diſpoſed in ſuch a manner as that the blood muſt have gone nearly in equal quantities from each ventricle into the aorta.

The edge of the opening in the ſeptum ventriculorum was exactly oppoſite to the centre of the aorta.

A Conformation of the Heart, ſo uncommon as in the preſent caſe, muſt neceſſarily lead thoſe who are acquainted with the laws of reſpiration and circulation to an explanation of many of the ſymptoms which this patient laboured under.

It is eaſy to be conceived, that in the caſe of this patient, the circulation could not be carried on regularly and perfectly. From the diminiſhed ſize of the lungs, it is probable that a very inadequate portion of blood circulated through them, and added to this, a large quantity of it neceſſarily paſſed through the foramen ovale and opening in the ſeptum ventriculorum without going through them at all. It alſo follows from the former circumſtance, that upon any exertion the heart would be overloaded and reſpiration performed very imperfectly, and hence thoſe diſtreſſing ſymptoms which continually accompanied her on the leaſt exertion, from the period at which ſhe began to walk till her death.

It has been before obſerved, that the edge of the opening in the ſeptum ventriculorum, was exactly oppoſite to the centre of the aorta, and was ſo ſituated as that the blood muſt have paſſed nearly in equal quantities from each ventricle into that veſſel. Now, as the right ſide of the heart is neceſſarily loaded under difficulty of breathing, a larger quantity of venous blood would, at that time, paſs into the aorta through the paſſage of communication common to the two ventricles, and through the foramen ovale, and thus account in a great meaſure for the degree of chillineſs ſhe experienced under all kind of exertion.

### Observations on Animal Heat.

Many important facts relative to reſpiration have, ſince very remote periods, been well inveſtigated and aſcertained. Since the more modern diſcoveries, however, many philoſophers have ingeniouſly explained ſome of the functions of the animal economy, and particularly that of the lungs, by reaſonings purely chemical. We cannot give a decided opinion upon theſe ſubjects, but we will, in as ſhort a manner as poſſible, preſent an outline of the latter, and attempt to ſhew that ſome difficulties will preſent themſelves to thoſe who conſider it too chemically.

Experiments fully demonſtrate, that the pure part of the air is materially altered by reſpiration. The capacity of this for heat far exceeds that of the expired gaſſes, from which it is evident that a conſiderable portion of heat has been, in ſome manner, diſpoſed of, during the contact of the inſpired air with the membrane which lines the air cells of the lungs.

The blood is formed from animal or vegetable matter, or from both, and, like each of them, abounds in hydrogen and carbon; both theſe bodies have a ſtrong affinity for the baſis of oxygen gas, and when they combine with it, caloric is always extricated.

According to Dr. Crawford, nearly one-ſixth of the oxygen gas inſpired, contributes to form aqueous vapour; the remaining five-ſixths enter into the compoſition of the expired carbonic acid gas. Now both theſe bodies being denſer than pure air, the laſt nearly in the proportion of 2 to 1, it is evident, that if a certain quantity of oxygen gas were to be inſtantly decompoſed by the carbon in the lungs to the production of carbonic acid gas, the heat that would be evolved from their difference of capacity, would be immediately ſet at liberty, and be productive of a very high degree of temperature, if it were not abſorbed by the blood at the inſtant of its liberation.

It appears that the inſpired air comes very nearly into contact tact with the blood contained in the minute ramifications of the pulmonary artery, in which it is highly charged with hydro-carbonaceous matter, and that the pure part of it* is decomposed by that combustible substance to the formation of aqueous vapour and of carbonic acid gas, the capacity of which being less than the capacity of oxygen gas, the superabundant heat immediately combines with the blood thus deprived of a portion of its carbone and hydrogen (and thus increased in capacity) to form arterial blood.

In the act of respiration then, the venous blood loses some combustible principles, by which its capacity becomes increased, and, at the same time, combines with the heat that results from the difference of capacity which exists between the inspired and the expired gasses.

In the course of circulation, especially through the capillary vessels, the blood continually takes up hydro-carbonaceous matter, by which its capacity for heat becoming diminished, sensible heat is gradually evolved, and the animal body thereby chiefly preserves its temperature.

When warm blooded animals are placed in a cold medium, their venous blood assumes a much darker hue than when they are placed in a warmer; for, as the animal heat greatly depends upon the evolution of the heat from the blood, in consequence of its combination with hydro-carbonaceous matter, more especially in the capillaries; so, therefore, when animals are placed in a cold medium, they deteriorate more air in a given time than when they are placed in a warmer; and hence it appears, that the quantity of heat which is separated from the air, and absorbed by the blood in the act of respiration, is, in every instance, and, cæteris paribus, proportioned to the necessity; there may then possibly be a certain degree of temperature at which arterial blood ceases to combine with the hydro-carbonaceous matter, and when there consequently cannot be a decomposition of pure air in the lungs, unless indeed it arises from the action of the chyle, which the blood acquires before it circulates through the lungs, and independently of that substance, which is probably chiefly secreted by the capillary vessels.

* According to some experiments which were made by the late Abbé Spallanzani, and which will be, or have been, published by Venturi, it appears that a great number of animals absorb nitrogene gas by the lungs from the respired air. A respected friend of mine has, likewise, (I believe) proved by experiment, that a certain volume of atmospherical air contained less nitrogene gas after he had respired it carefully for some time, than it did before the commencement of the experiment.

The rarity of the air in tropical, and its denfity in polar, climates might be adduced as a caufe of the permanency and nearly equal degrees of animal heat in thofe fituations; but if this were the cafe, a highly carburetted ftate of the arterial blood would of neceffity be found in the former, which I believe is not the fact.

It is rather extraordinary that the animal heat, in the cafe before us, was not confiderably lefs than has been noticed. It is, however, probable that the blood was more completely carburetted in the act of circulation, than if the ufual circumftances had exifted (for when any part of the fyftem is affected, the powers of life are often præternaturally exerted in another connected with it): and if this conjecture be allowed, a comparatively greater proportion of the heat, which was imparted to the blood in the lungs, muft neceffarily have been feparated in the body.

The oxydation of the blood is a fubject ftill involved in fome obfcurity. It cannot, I think, be afcertained, whether the bafis of oxygen gas combines with the blood in the act of refpiration, and afterwards circulates with it in a chemically combined ftate, unlefs it is proved that the quantity of it confumed in that procefs exceeds that which forms one of the component parts of the aqueous vapour and carbonic acid gas expired: and, if the proportion of oxygen in the two latter be greater than that which is infpired, another fource of difficulty will again prefent itfelf; for, we have no method of diftinguifhing that aqueous vapour which, it is fuppofed, is formed by the combination of the bafis of oxygen gas, and a portion of the hydrogen of the blood, and that which fome of the veffels fituated on the membrane which lines the cells of the lungs fecrete, in common with thofe of other membranous furfaces. Lavoifier and Seguin have made fome experiments, from which they were induced to believe, that the quantity of oxygen gas confumed in refpiration was greater than that which enters into the compofition of the gaffes and vapour expired; but fome doubts concerning this important fubject feem ftill to remain with fome eminent phyfiologifts.

The confideration of the placental circulation, and of the change which is effected on the blood contained in thofe minute branches of the umbilical arteries which ramify upon the membrane lining the cells of the maternal part, would, however, rather feem to favour the idea of oxydation. It is well known that the maternal and fœtal fyftems cannot be injected by one another, and that the blood of the umbilical vein is more florid than that in the umbilical arteries. Now, if it could be proved that the extremities of the umbilical vein took up the blood from from the materna part of the placenta, a good reafon might be affigned for the change of colour; but this has not been proved, and analogy goes againft it. The change therefore, probably, either depends upon fomething which is taken up by the veffels, and combines with the blood contained in them; or, it may more probably take place in the fame manner as in ordinary refpiration; but with this difference, that the maternal blood itfelf (on a fuppofition that it has combined with oxygen in the lungs) imparts a portion of it to the blood in the fœtal veffels, either at minute open mouths, or by paffing currents, and confequently through the coats of the veffels and the membrane of the cells.

Some experiments, however, at which I affifted, fome time ago, in fome degree led me to fuppofe that oxygen does not combine with the blood in the act of refpiration. Several animals were confined in a certain quantity of hydrogen gas (which is not immediately fatal to life) which had been tefted with nitrous gas, and afterwards expofed, for fome time, to a mixture of lime and lime water. The animals remained under expofure a fufficient time to fhew whether carbonic acid gas had been expired; but there was not found a larger portion in the hydrogen gas after the experiment than could be accounted for from the action of the atmofpherical air which remained in the lungs of the animals after the expofure. If a portion of the expired carbonic acid gas were formed in the courfe of circulation, in confequence of the oxydation of the blood in the lungs, we ought neceffarily to have detected a larger quantity of it; for the blood, in this cafe, muft have been highly charged with oxygen at the time of the expofure; and the hydrogen gas, fo far as I know, could not have prevented the free exit of carbonic acid gas from the extremities of the pulmonary veins.

Although animal heat has of late been principally (if not altogether) attributed to the decompofition of venous blood in the lungs, as before ftated; yet many phænomena which take place in the living body, certainly ftagger opinion in this refpect. It appears from many circumftances extremely probable, that caloric is received into the fyftem, and feparated from it in various other proceffes, more particularly perhaps during the digeftion of our aliment. It is likewife very probable that heat is evolved during the different fecretions from the blood; and that the conftant combinations and productions of new fluids by the glands, conftitute a grand fource of animal heat.

Many of the phænomena of animal life have been lately attributed to the influence of the oxygen taken into the body in the act of refpiration; that bafis, according to many philofophers,

 effects effects and explains many of thoſe proceſſes which will perhaps ever ſurpaſs the limits of human underſtanding. The laws of irritation, ſenſation, volition, &c. have been explained by the ſuppoſed influence of oxygen in the ſyſtem. I cannot inveſtigate a ſubject ſo abſtruſe; but no concluſion ought to be drawn which is not fully warranted by experiment. In the progreſs of inveſtigation, we ſhould proceed from known facts to what is ſtill unknown; we ſhould ever be cautious how we apply chemical theories to the living body. I cannot deny the wonderful effects which have been attributed to oxygen; but until the principle of life is better known, we ſhall perhaps fruitleſsly attempt the ſolution of many phænomena by the prevailing theories of the day, and thus gradually add more and more to the already inexhauſtible ſtock of ſpeculations.

I have endeavoured to be as clear and conciſe as the limits of theſe Obſervations could admit, and have purpoſely avoided all calculations reſpecting the quantities of carbon and oxygen in carbonic acid gas, as well as a ſtatement of the accurate differences in the capacities for heat of the gaſſes, the venous and the arterial blood. I have likewiſe intentionally waved the conſideration of the oxydation of the blood out of the body, which does not appear to be ſtrictly analogical, when it is compared with the perſpiratory proceſs.

Is the change which the blood undergoes in the lungs in the act of reſpiration referable to a chemical operation taking place in that viſcus? If it be, many difficulties will ariſe when we ſhall attempt the explanation. The principle of life acts where this proceſs is accompliſhed; and can any one poſitively aſſert, that chemical laws have dominion in the living body? If that revolution which Chemiſtry has lately under-[illegible]one, had not been carried on and directed by men, who to the greateſt ſcience added a moſt profound knowledge of the animal economy, our ſchools would already have reſounded with the extravagancies of a Paracelſus, which it is to be feared a wrong application of the beautiful ſcience of Chemiſtry would very ſoon again revive.

All the truly uſeful and ſcientific knowledge we can ever hope to gain, can only be had by obſervation and experiment; many of very ſuperior intellect have already been ſo prone to theoretical ſpeculations as to materially injure the cauſe which they meant to improve: inſtead, therefore, of wholly giving up our time to indulging ingenious fancies, let a portion of it be devoted to the relation of ſimple facts; every thing elſe, [illegible]ing the fruit of ſpeculation, muſt ever be liable to over-[illegible]w.

